# Chemotherapeutic effect of a novel temozolomide analog on nasopharyngeal carcinoma in vitro and in vivo

**DOI:** 10.1186/s12929-015-0175-6

**Published:** 2015-08-19

**Authors:** Thomas C. Chen, Hee-Yeon Cho, Weijun Wang, Stephanie J. Wetzel, Anupam Singh, Jenny Nguyen, Florence M. Hofman, Axel H. Schönthal

**Affiliations:** Department of Neurosurgery, University of Southern California, Los Angeles, CA USA; Department of Molecular Microbiology & Immunology, University of Southern California, Los Angeles, CA USA; Department of Pathology, University of Southern California, Los Angeles, CA USA

**Keywords:** Nasopharyngeal carcinoma, O6-methylguanine-DNA methyltransferase, Perillyl alcohol, Temozolomide, Chemoresistance

## Abstract

**Background:**

Many patients with nasopharyngeal carcinoma (NPC) face poor prognosis. Due to its hidden anatomical location, the tumor is usually diagnosed quite late, and despite initially successful treatment with radiation and cisplatin, many patients will relapse and succumb to the disease. New treatment options are urgently needed. We have performed preclinical studies to evaluate the potential NPC therapeutic activity of a newly developed analog of temozolomide (TMZ), an alkylating agent that is the current chemotherapeutic standard of care for patients with malignant glioma.

**Results:**

TMZ was covalently conjugated to the natural monoterpene perillyl alcohol (POH), creating the novel fusion compound NEO212. Its impact on two NPC cell lines was studied through colony formation assays, cell death ELISA, immunoblots, and in vivo testing in tumor-bearing mice. In vitro, NEO212 effectively triggered tumor cell death, and its potency was significantly greater than that of its individual components, TMZ or POH alone. Intriguingly, merely mixing TMZ with POH also was unable to achieve the superior potency of the conjugated compound NEO212. Treatment of NPC cells with NEO212 inactivated the chemoprotective DNA repair protein MGMT (O6-methylguanine methyltransferase), resulting in significant chemosensitization of cells to a second round of drug treatment. When tested in vivo, NEO212 reduced tumor growth in treated animals.

**Conclusion:**

Our results demonstrate anticancer activity of NEO212 in preclinical NPC models, suggesting that this novel compound should be evaluated further for the treatment of patients with NPC.

**Electronic supplementary material:**

The online version of this article (doi:10.1186/s12929-015-0175-6) contains supplementary material, which is available to authorized users.

## Background

Nasopharyngeal carcinoma (NPC) is a tumor arising from the epithelial cells of the nasopharynx, which is located behind the nose and above the back of the throat. NPC differs significantly from other cancers of the head and neck, based on its causes, occurrence, clinical behavior, and treatment options. It is uncommon in many parts of the world, but is endemic in the Mediterranean basin, Alaska, Southeast Asia, the Guangdong province of southern China, and Taiwan [1, 20, 29].

NPC’s difficult-to-observe location behind the nose, where the nasal passages and auditory tubes join the remainder of the upper respiratory tract, create two major hurdles for effective therapy. First, due to its hidden location, NPC is usually diagnosed quite late during its development and in fact, the diagnosis is often made by lymph node biopsy, when the primary cancer already has begun to spread to other parts of the body. Second, the anatomical position of the primary tumor is not easily amenable for surgery and local control, which is why biopsies are performed on cervical lymph node metastases, rarely on the primary tumor [19].

Treatment of NPC usually involves radiation therapy, cisplatin-based chemotherapy, or a combination of the two. Surgery, which is rarely used, may be applied to remove cancerous lymph nodes in the neck area; radical resection is often ineffective, and extremely disfiguring. Radiation treatment often leads to esophagitis and impaired swallowing. Cisplatin-based chemotherapy is often tolerated for only two or three cycles. The exact treatment plan is devised based on several factors, including stage of the cancer, overall health of the patient, and the extent of side effects the patient is willing to tolerate. However, despite initially successful treatment, many patients will relapse after treatment, and management of NPC remains one of the biggest clinical challenges. Overall survival after recurrence is quite poor with median survival ranging from 7–22 months. Therefore, more effective and better-tolerated treatment options for NPC are desperately needed [1, 13, 27, 29].

We therefore performed preclinical studies to determine the potential NPC therapeutic efficacy of a novel analog of the alkylating agent temozolomide (TMZ). TMZ is the standard of chemotherapeutic care for patients with glioblastoma multiforme (GBM) and melanoma [31]. In both applications however, the emergence of treatment resistance is common and is frequently linked to the overexpression of MGMT (O6-methyl-guanine DNA methyltransferase), a DNA repair protein that removes alkyl groups located at the O6-position of guanine [7, 22]. This repair process is quite specific and therefore provides protection primarily against alkylating chemotherapeutics that target O6-guanine, such as temozolomide, mitozolomide, dacarbazine, or fotemustine [7, 14, 25]. MGMT activity is unusual in that it represents a “suicide mechanism”, whereby acceptance of the alkyl group from DNA irreversibly inactivates the enzyme and leads to its rapid degradation [22]. This feature is exploited by the use of specific MGMT inhibitors, such as O6-benzylguanine (O6BG), which act as pseudosubstrates [15]. Benzylation of MGMT via reaction with O6BG causes the same structural change in the enzyme as that seen after alkylation following DNA repair, and therefore also leads to rapid degradation of the protein [23].

We have developed a novel analog of TMZ, where the monoterpene perillyl alcohol (POH) was covalently conjugated to TMZ via a carbamate bridge. POH is a natural constituent of lavender, lilac oil, cherries, spearmint, celery seeds, and certain other plants [28]. It has shown promising activity in several mouse tumor models [11, 21], and clinical trials performed in Brazil demonstrated that it exerted therapeutic activity against recurrent glioblastoma when it was administered to patients via nasal inhalation [5, 9]. Based on these promising results, we hypothesized that covalently linking POH to TMZ might yield a compound with inherently increased anticancer activity. Here we present evidence that this novel chimeric molecule, which we termed TMZ-POH or NEO212, displays significant activity in preclinical models of NPC, and therefore has promise for clinical evaluation in this tumor type.

## Methods

### Pharmacological agents

NEO212 was provided by NeOnc Technologies (Los Angeles, CA) and was dissolved in DMSO (dimethyl sulfoxide) at 100 mM. TMZ was obtained from the pharmacy at the University of Southern California (USC) and dissolved in DMSO to a concentration of 50 mM. POH, O6BG, cisplatin, MG-132, and DMSO were purchased from Sigma-Aldrich (St. Louis, MO). In all cases of cell treatment, the final DMSO solvent concentration in the culture medium never exceeded 1 %, and was much lower in most cases. Stock solutions of all drugs were stored at −20 °C.

### Cell lines

Human NPC cell lines TW1 and TW4 were propagated in DMEM supplemented with 10 % fetal bovine serum (FBS), 100 U/mL penicillin, and 0.1 mg/mL streptomycin in a humidified incubator at 37 °C and a 5 % CO_2_ atmosphere. All cell culture reagents were provided by the Cell Culture Core Lab of the USC/Norris Comprehensive Cancer Center and prepared with raw materials from Cellgro/MediaTech (Manassas, VA); FBS was obtained from Omega Scientific (Tarzana, CA). Both NPC cell lines were kindly provided by Prof. Chin-Tarng Lin (Taiwan University, Taiwan) [17].

### Colony formation assay

From 600 to 800 cells were seeded into each well of a 6-well plate and treated as described previously [4]. After 48 h of drug exposure, medium was replaced with fresh medium without drugs. The exception was O6-benzylguanine, which was added at time 0 h and again at 48 h (continuous exposure to this agent did not exert cytotoxic effects). Newly formed colonies (>50 cells) were stained after 12–14 days. Each assay was repeated at least once.

### Cell death ELISA

Ten thousand cells were seeded into each well of a 96-well plate. The next day, increasing concentrations of drug, or vehicle only, were added. After 24 h of incubation, cell cultures were analyzed for the extent of apoptosis by using the Cell Death Detection ELISA^PLUS^ (Roche Life Science, Indianapolis, IN) according the manufacturer’s instructions.

### Immunoblots

Total cell lysates were analyzed by Western blot analysis as described earlier [24]. The primary antibodies were purchased from Cell Signaling Technology (Beverly, MA), Santa Cruz Biotechnology, Inc. (Santa Cruz, CA), or Abcam (Cambridge, MA) and used according to the manufacturers’ recommendations. All immunoblots were repeated at least once to confirm the results.

### Animal model

All animal protocols were approved by the Institutional Animal Care and Use Committee (IACUC) of USC, and all rules and regulations were followed during experimentation on animals. Athymic mice (Harlan, Inc., Indianapolis, IN) were implanted into the right flank with 2x10^6^ TW4 cells harboring the luciferase gene, so that tumor growth could be monitored via bioluminescence imaging. Approximately ten days later, once palpable tumors had developed, six animals were assigned to different treatment groups. The control group received vehicle only (45 % glycerol, 45 % ethanol, 10 % DMSO), whereas the treatment groups received NEO212 dissolved in vehicle. Tumor volume was measured with calipers every 2–3 days. In parallel, body weight was recorded.

### Statistical analysis

All parametric data were analyzed using the Student *t*-test to calculate the significance values. A probability value (*p*) <0.05 was considered statistically significant.

## Results

### Cytotoxic effects of NEO212 on NPC in vitro

**Fig. 1:**
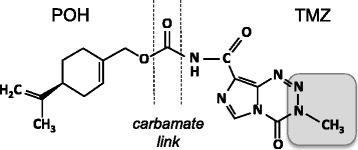
Chemical structure of NEO212. Shown is the carbamate linkage between the monoterpene perillyl alcohol (POH) and the amide functional group of the imidazotetrazine TMZ. Shaded area: TMZ moiety that forms the highly reactive methyldiazonium ion that methylates DNA after spontaneous decomposition of the pro-drug TMZ that takes place in aqueous solution at neutral pH [31]

**Fig. 2:**
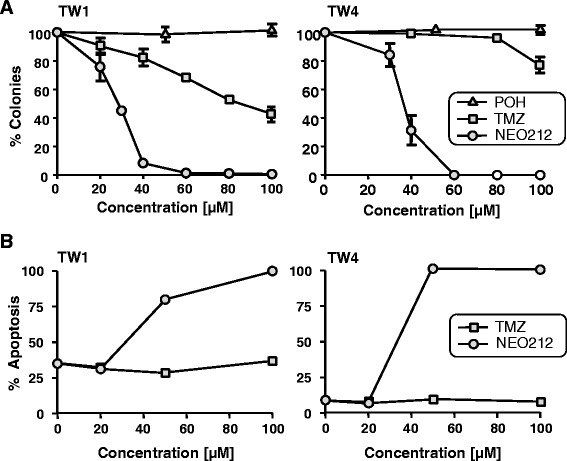
Survival of NPC cells after drug treatment with NEO212, TMZ or POH. **a**, TW1 and TW4 cells were exposed to increasing concentrations of NEO212 (circles), TMZ (squares) or POH (triangles) for 48 h, and survival was determined via colony formation assay (CFA). The number of colonies formed by untreated cells was set at 100 %; ±SD, n ≥ 3. **b**, Apoptosis of TW1 and TW4 cells after treatment with increasing concentrations of NEO212 (circles) or TMZ (squares), as measured by cell death ELISA assay (averages from 2 experiments)

A novel analog of temozolomide (TMZ) was created by covalently linking perillyl alcohol (POH) to TMZ’s amide functionality (Fig. 1). The cytotoxic potency of this new compound, called NEO212, was analyzed by colony formation assay (CFA) in two different human NPC cell lines (TW1 and TW4) and compared to the cytotoxic effects of the individual parent compounds, TMZ and POH. As shown in Fig. 2a, NEO212 potently suppressed colony formation in both cell lines, with IC50s of 32 and 37 μM in TW1 and TW4 cells, respectively. In comparison, TMZ was significantly weaker, with IC50s of 85 and >100 μM. POH did not display any cytotoxic effects at concentrations up to 100 μM, consistent with published observations [6, 30] that this compound generally requires concentrations in the millimolar range to trigger cell death.

Potent induction of NPC cell death by NEO212 was further confirmed with the use of a cell death ELISA assay, specifically measuring the extent of drug-induced apoptosis. Here the cytotoxic differential between NEO212 and TMZ became even more pronounced. While 50 μM NEO212 caused very extensive apoptosis in both TW1 and TW4 cell cultures, TMZ up to 100 μM exerted only marginal effects (Fig. 2b).

**Fig. 3:**
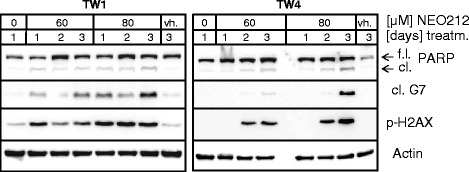
Expression levels of markers of DNA damage and apoptosis. TW1 and TW4 cells were treated with 60 and 80 μM NEO212, and cellular lysates were harvested 1, 2 or 3 days later. In parallel, untreated cells were harvested after 1 day, and vehicle-treated cells (vh.) were collected after 3 days. Western blot analysis was performed with antibodies specific for full-length (f.l.) and cleaved (cl.) PARP, cleaved caspase 7, and p-H2AX, with actin as the loading control

We next characterized the effects of NEO212 at the molecular level by performing Western blot analysis of two types of protein markers: (i) caspase 7 and PARP (poly ADP-ribose polymerase-1); proteolytically cleaved smaller forms indicate ongoing apoptosis; and (ii) phosphorylated histone H2A (γ-H2AX) protein, an established marker for double-strand DNA breaks. TW1 and TW4 cells were treated with NEO212 and harvested after 24, 48, and 72 h. As shown in Fig. 3, treatment with NEO212 caused DNA damage and triggered apoptosis, as indicated by greatly increased expression levels of γ-H2AX and the emergence of cleaved PARP and cleaved caspase 7. Overall, drug effects were somewhat stronger in TW1 as compared to TW4 cells, in keeping with the slightly lower IC50 of NEO212 that was observed in the CFAs of TW1 cells above. Combined, these results establish that NEO212 exhibits potent cytotoxic effects against NPC in vitro, and these effects were stronger than those of TMZ or POH. Overall, NEO212 acted more quickly and with greater intensity.

**Fig. 4:**
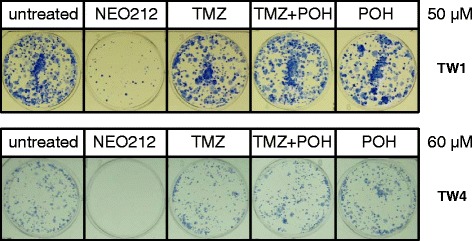
Colony formation after combination treatment. TW1 and TW4 cells were exposed to 50 or 60 μM each of NEO212, TMZ, or POH individually, as well as to TMZ mixed with POH (TMZ + POH, each at either 50 or 60 μM), and colony-forming ability was determined. Shown are photographic images of colonies formed in each well, which represent highly representative results from several repeats of this experiment. Note that equimolar mixes of TMZ + POH are unable to achieve the superior cytotoxicity of the conjugated compounds in NEO212

Because NEO212 represents a chimeric structure consisting of covalently linked TMZ and POH, we next investigated whether merely mixing the two parental compounds would be able to achieve the superior cytotoxicity of the conjugated molecule. TW1 and TW4 cells were exposed to NEO212, TMZ, POH, or TMZ mixed with POH (TMZ + POH) and long-term cell survival was analyzed by CFA. However, as shown in Fig. 4, the individual compounds mixed together at equimolar concentrations were unable to achieve the strong inhibitory effect of NEO212. Thus, these results present NEO212 as a novel compound with increased potency over TMZ that cannot be matched by merely mixing its individual parts, TMZ and POH.

### Influence of MGMT on NPC cell killing by NEO212

**Fig. 5:**
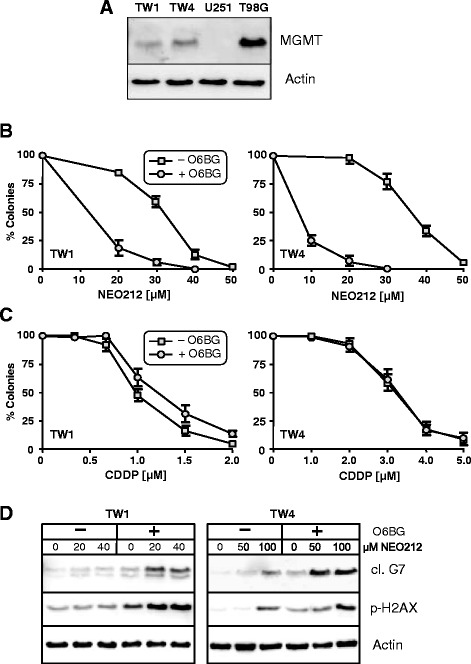
Role of MGMT in cytotoxic effects of NEO212. **a**, MGMT basal expression levels in TW1 and TW4 cells, compared to U251 and T98G glioblastoma cell lines, which are known to be negative and positive, respectively, for MGMT protein expression. Shown is Western blot analysis with actin as the loading control. **b**, TW1 and TW4 cells were pre-treated with or without 15 μM O6BG for 1 h, followed by the addition of increasing concentrations of NEO212 for 48 h. **c**, Cells were pre-treated with O6BG as in B, followed by the addition of cisplatin (CDDP) for 48 h. In both B and C, the ability of cells to survive and form colonies was determined. Number of colonies from untreated cells was set at 100 % (±SD, *n* = 3-4). **d**, Cells were treated as in B, and after 72 h of incubation the cells were harvested for Western blot analysis of cleaved (cl.) caspase 7 (marker of apoptosis) and p-H2AX (marker of DNA damage). Actin was used as the loading control

The DNA repair protein MGMT is known to play a key role in cellular resistance to TMZ; we therefore investigated whether it would impact the cytotoxic potency of NEO212 in NPC. First, we determined the basal expression level of MGMT by Western blot analysis of TW1 and TW4 cells and compared it to MGMT levels in two well-characterized glioblastoma cell lines, U251, which is negative, and T98G, which is strongly positive. As shown in Fig. 5a, both NPC cell lines were positive for MGMT expression; TW4 cells had somewhat higher levels than TW1 cells, but overall neither of them was quite as strongly positive as T98G cells.

O6-benzylguanine (O6BG) is an inhibitor of MGMT, known to sensitize MGMT-positive cells to alkylating agents, such as TMZ. We exposed TW1 and TW4 cells to O6BG, followed by treatment with increasing concentrations of NEO212. Long-term cell survival was determined by CFA. As shown in Fig. 5b, pretreatment of NPC cells with O6BG resulted in pronounced increase in cytotoxic efficacy of NEO212, indicating that the moderate MGMT protein levels in these cells were chemoprotective against NEO212. We also performed this experiment with cisplatin, a commonly used chemotherapeutic agent for NPC and known DNA crosslinking agent. In this case however, combination with O6BG did not further increase cisplatin cytotoxicity in either NPC cell line; rather, in TW1 cells there was a minor antagonistic effect (which was not statistically significant) (Fig. 5c). Combination effects of O6BG and NEO212 were also investigated by Western blot for the expression levels of γ-H2AX (DNA damage marker) and cleaved caspase 7 (apoptosis marker). It emerged that NEO212 triggered both processes more strongly after pretreatment with O6BG (Fig. 5d). Altogether, these results indicated that NEO212 had retained the alkylating function of its parent molecule TMZ. Although NEO212’s cytotoxic efficacy was substantially greater than that of TMZ, MGMT expression levels in these NPC cells still reduced the compound’s overall impact to some extent.

**Fig. 6:**
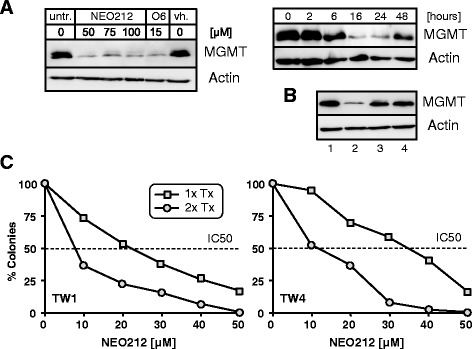
Effects of NEO212 on MGMT expression and repeated treatments. **a**, Left panel: TW4 cells were treated with 50, 75, and 100 μM NEO212, 15 μM O6BG (O6), or vehicle (vh.) for 24 h. Control cells received no treatment. Right panel: TW4 cells were treated with 50 μM NEO212 for the indicated time periods. In all cases, cell lysates were prepared and analyzed by Western blot with antibodies specific to MGMT or actin (as a loading control). **b**, TW4 cells were treated with 1: vehicle only; 2: 50 μM NEO212; 3: 50 μM NEO212 combined with 5 μM MG-132 (a proteasome inhibitor); 4: 5 μM MG-132. After 16 h, cells were harvested and analyzed by Western blot. **c**, Cells were treated with increasing concentrations of NEO212 at time 0 h (1x Tx). After 24 h, some of the cells received a second dose of the same NEO212 concentration (2x Tx). The number of formed colonies was determined 10 days later. Dotted line marks IC50 values, which shifted to the left upon repeat treatment

Because MGMT protein levels exerted a noticeable influence on NEO212’s cytotoxic potency, we next investigated whether NEO212 affected MGMT expression over time. As shown in Fig. 6a, this was indeed the case. Treatment with 50 μM NEO212 effectively diminished the levels of MGMT protein, and this inhibition was as strong as the inhibition achieved with the potent MGMT-inhibitor O6BG. Downregulation of MGMT became apparent as early as 6 h after the addition of NEO212 to the cell cultures and was most pronounced at 16 to 24 h. This effect was transient, because at 48 h MGMT levels appeared to slowly recover. Diminished MGMT protein levels in response to NEO212 treatment were the result of accelerated proteasomal degradation, as indicated by the inclusion of MG-132, a potent proteasome inhibitor, which prevented MGMT downregulation (Fig. 6b).

The strong downregulation of MGMT levels induced by NEO212 was intriguing, because it suggested these cells might now have become sensitized to further treatment with alkylating agents. To test this prediction, we performed a repeat drug treatment, where NEO212-treated cells received a second dose of NEO212 24 h after the first. Figure 6c presents the outcome of this approach with the use of long-term CFAs. It shows that the addition of a second dose of NEO212 after 24 h resulted in considerably enhanced cell death. Repeat treatment reduced the IC50 from 22 to 8 μM in TW1 cells, and from 35–11 μM in TW4 cells. This result is of particular interest, because it would support a continuous once-daily dosing regimen of NEO212 in vivo and/or in the clinic, as is currently done for glioblastoma patients [26].

### Effects of NEO212 in vivo

Lastly, we investigated whether NEO212 exerted anticancer activity against NPC in vivo. To address this issue, TW4 cells were transfected with the firefly luciferase gene and then implanted into the flanks of 12 nude mice. After palpable tumors had developed, all animals were imaged to confirm tumor take, and based on the extent of luminescence signals the animals were separated into two groups, so that each group had similar representation of the slightly variable tumor sizes. Thereafter, animals received either vehicle or NEO212 once daily for 10 days, and tumor development was monitored by performing regularly scheduled bioluminescent whole-body imaging.

**Fig. 7:**
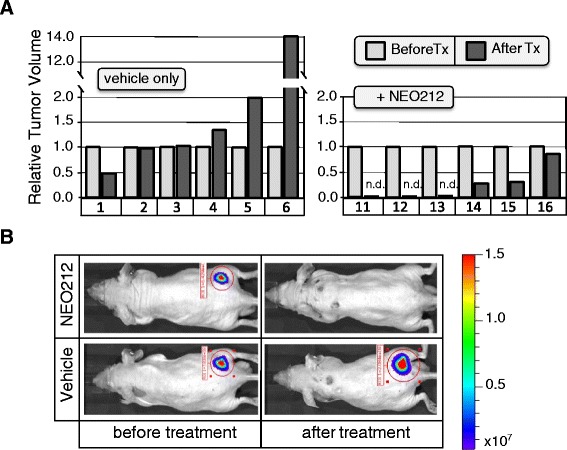
Antitumor effects of NEO212 in vivo. Tumor-bearing animals received vehicle or 30 mg/kg NEO212, given by subcutaneous injection into the neck area (distal to the site of tumor location in the flank), once daily over the course of 10 days. Twelve days after termination of treatment, tumor growth was compared among all animals via whole-body bioluminescent imaging. **a**, Shown is the relative change in bioluminescent radiance for each animal from the time treatment was started (set at 1.0) until 22 days later. Animals 1 through 6 had received vehicle only, whereas animals 11 through 16 had received NEO212. **b**, Two individual animals are shown as examples: one animal (#4) from the vehicle-treated group and one animal (#3) from the NEO212-treated group. Animal #13 (similar to animals 11 and 12) presented with undetectable tumor. The difference in bioluminescent radiance between the group of NEO212-treated animals and the group of vehicle-treated animals was statistically significant (*p* > 0.01)

Altogether, treatment of tumor-bearing mice with NEO212 resulted in obvious anticancer outcomes. Figure 7a shows the results for all 12 animals. Overall, in vivo growth of TW4 cells was fairly slow and not particularly aggressive. Still, among the group of vehicle-treated animals, three mice showed an increase in bioluminescent signal by 1.35-, 2-, and 14-fold as compared to before treatment; two animals showed no difference before and after, and one animal presented with 50 % reduction in tumor signal. In comparison, none of the six animals treated with NEO212 had increased bioluminescent tumor signal; rather, the signal disappeared entirely in three animals, and the other three presented with signal reductions of 15, 65, and 70 %. Figure 7b presents imaging results from two representative mice, where one had received vehicle only, and the other NEO212. As can be seen, tumor size in the vehicle-treated animal had increased as compared to before treatment. In contrast, there was no detectable tumor signal in the animal treated with NEO212.

In our in vivo experiments, body weight of animals was closely monitored. There was no major loss of weight during the 10-day course of drug treatment or thereafter (not shown). At the end of the experiment (12 days after the termination of drug treatment), all six vehicle-treated and three NEO212-treated animals were euthanized for histopathological analysis. However, no signs of potentially toxic impact of NEO212 on intestines, liver, kidney, lung, or brain could be detected (Additional file 1: Figure. S1). As well, three of the NEO212-treated animals were kept for an additional month after cessation of treatment under continuous observation; these animals continued to thrive and no noticeable long-term toxic drug effects became apparent. In addition, we treated a set of non-tumor-bearing mice with daily NEO212 at 30 and 60 mg/kg for 7 days, and then performed a liver function test and a complete blood count (CBC) with differential. Here as well, no signs of toxicity emerged; there was no indication of impairment of liver function (Additional file 1: Table S1), nor were there any significant changes in the absolute or relative numbers of red or white blood cells, hematocrit, or hemoglobin (Additional file 1: Tables S2, S3). Altogether, these outcomes indicated that NEO212 was very well tolerated by these animals.

## Discussion

For current practice, NPC patients are treated with radiation therapy or combined therapy of radiation and cisplatin-based chemotherapy [1, 29]. However, radiation therapy often leaves the patients with severe symptoms of esophagitis and difficulty swallowing; cisplatin-based chemotherapy is often poorly tolerated, with common side effects including nephrotoxicity, ototoxicity and myelotoxicity, where most patients can only tolerate several cycles before it needs to be stopped [18]. In order to explore alternative, better-tolerated therapeutic approaches, we have performed preclinical studies to evaluate the potential NPC therapeutic activity of the novel compound NEO212. NEO212 was generated by covalently conjugating the natural monoterpene perillyl alcohol (POH) to the alkylating agent temozolomide (TMZ). Both of these components, individually, have been known to exert therapeutic activity in cancer patients. TMZ has become the current standard of chemotherapeutic care for patients with malignant glioma, and it is also frequently used in cases of advanced melanoma [31]. In phase I/II clinical studies performed in Brazil, intranasal inhalation therapy with POH has demonstrated well-tolerated clinical activity in patients with recurrent malignant glioma [8, 9], but this treatment regimen has not yet entered common clinical practice.

Our studies presented here demonstrate that the conjugated compound NEO212 exerts potent anticancer activity that is significantly greater than the activity of its individual parts, TMZ or POH. When tested in vitro in two different NPC cell lines, the cytotoxic potency of NEO212 was approximately 3-fold greater than that of TMZ, and about 25-fold greater than that of POH (Fig. 2 and not shown). In particular, within the concentration range of 40–60 μM, where NEO212 killed 100 % of NPC cells, TMZ exerted hardly any cytotoxic activity, and POH was completely ineffective (Figs. 2 and 4). Intriguingly, merely mixing the two individual agents, TMZ and POH at equimolar concentrations, was unable to achieve the superior activity of the conjugated molecule. For instance, while 50 μM NEO212 dramatically reduced colony formation of NPC cells, treatment of cells with 50 μM TMZ mixed with 50 μM POH had no substantial activity (Fig. 4). Taken together, these results not only present NEO212 as a compound with cytotoxic activity against NPC cells, but also indicate that this conjugate represents a novel chemical entity with inherently increased potency that is greater than the sum of its parts.

It is not yet entirely clear why the conjugated molecule is substantially more potent than the sum of its parts. TMZ is a well-characterized alkylating agent that exerts toxic activity primarily via its methylation of the O6-position of guanine, which results in double-strand DNA breaks that trigger cell death. Cells that have high levels of the DNA repair protein MGMT are protected against TMZ, because MGMT removes methyl groups from O6-guanine, thereby reversing the lesion set by TMZ [14, 22, 25]. We have analyzed the expression levels of MGMT in the two NPC cell lines used in our study, and have documented significant amounts of this DNA repair protein in both cell lines. While the presence of MGMT suffices to explain why these cells are resistant to TMZ, NEO212 nonetheless unfolded its cytotoxic potency against these cells. This latter finding is particularly relevant, because elevated expression of MGMT can be expected in the majority of NPCs [16]. Nonetheless, while NEO212 exerts its activity even against MGMT-positive cells, the inclusion of O6BG, which eliminates MGMT function, further increases its cytotoxic impact (Fig. 5). This latter finding not only indicates that at least part of NEO212’s toxic effect is mediated via methylation of O6-guanine, but it also suggests that patients with MGMT-negative tumors should be particularly responsive to NEO212 treatment.

While using O6BG, we also tested the effects of cisplatin, representing the commonly used chemotherapy for patients with NPC. While both NPC cell lines were responsive to cisplatin with cytotoxic IC50s in the range of 1–3 μM, the inclusion of O6BG did not further increase cell killing by cisplatin (Fig. 5). Rather, in the case of TW1 cells, there was a tendency for slightly increased survival in the presence of O6BG. The inability of O6BG to enhance killing by cisplatin was somewhat expected, because the repair effect of MGMT is known to be selective for alkylated O6-guanine [7, 14, 22], and cisplatin rather acts as a DNA crosslinking agent [2]. However, in contrast to our results, a very recent study by Chen et al. [3] did show that inclusion of O6BG resulted in increased cisplatin cytotoxicity in NPC cell lines HONE-1 and TW1 in vitro. The reasons for this obvious discrepancy are unknown and further studies are needed to resolve this conflicting issue.

With regard to the molecular functions of NEO212, we observed that this compound was able to down-regulate MGMT protein levels in vitro (Fig. 6a). This inhibitory effect was very pronounced within 16–24 h after the onset of treatment, but in the absence of additional treatment MGMT levels began to recover again at around 48 h. MGMT down-regulation appeared to be mediated at the post-translational level via accelerated degradation of MGMT protein, because inclusion of the proteasome inhibitor MG-132 prevented this effect (Fig. 6b), consistent with the well-described “suicide mechanism” by which MGMT protein is known to repair O6-methylguanine lesions [22].

In the context of MGMT’s chemoprotective activity, our finding that NEO212 minimized MGMT protein levels was intriguing, because it suggested that repeated drug treatments might be more effective because less MGMT is present to neutralize DNA alkylation. This hypothesis was put to the test by exposing drug-treated cells to a second round of NEO212 treatment. Our results show that this approach lowered the IC50 of NEO212 even further into the 10-μM range (Fig. 6c). While we don’t yet know clinically relevant concentrations of NEO212, those of TMZ may serve as an orientation. The peak concentrations of TMZ measured in the plasma of human cancer patients are 50–70 μM [10, 12]. These values are lower than the IC50 we measured for either NPC cell line when treated with TMZ (80 to >100 μM, Fig. 2), indicating that TMZ would not be effective in NPC patients. In comparison, it is conceivable, based on its chemical structure and its predicted physicochemical properties, that NEO212 might achieve plasma concentrations that are similar to TMZ, if not higher. In our in vitro experiments, the IC50 of NEO212 is in the 20–35 μM range and becomes even lower (10 μM) after repeat treatment. Together, these considerations provide reasonable support to predict that NEO212 will exert therapeutic activity in patients with NPC.

We furthermore observed that NEO212 exerted activity against xenotransplanted NPC cells in mice. While TW4 cells implanted into the flanks of mice did not reveal very aggressive growth, they nonetheless caused efficient, slow-growing tumor formation that was significantly (*p* < 0.01) reduced by a 10-day course of treatment with NEO212. This positive outcome is particularly interesting in view of the MGMT-positive status of these tumor cells, because it indicates that NEO212 can reach concentrations in vivo that are sufficiently high to exert therapeutic activity.

## Conclusions

Current therapy for NPC patients involves radiation therapy, which may be combined with cisplatin-based chemotherapy. Overall however, this treatment approach oftentimes is poorly tolerated and, despite initially successful treatment, many patients will relapse. In comparison, our study demonstrates effective anticancer activity of NEO212 in vitro and in vivo, at concentrations that are expected to be achievable in patients. We therefore propose that NEO212 should be investigated further as a potentially more effective and better-tolerated therapy for NPC patients.

## Additional file

Additional file 1: Table S1. Results from liver function test (SuperChem). **Table S2.** Results from complete blood count (CBC). **Table S3.** Results from complete blood count (differential). **Figure S1.** Histopathological organ analysis. (PDF 6162 kb)

## Abbreviations

CFA: Colony formation assay
MGMT: O6-methylguanine-DNA methyltransferase
O6BG: O6-benzylguanine
POH: Perillyl alcohol
TMZ: Temozolomide
NEO212: Perillyl alcohol covalently linked to temozolomide (TMZ-POH)
