# Supplementary material for: Chemotherapeutic effect of a novel temozolomide analog on nasopharyngeal carcinoma in vitro and in vivo
Source: J Biomed Sci. 2015 Aug 19;22(1):71. doi: 10.1186/s12929-015-0175-6 (PMC4539921; doi:10.1186/s12929-015-0175-6)

SUPPLEMENTARY DATA (Tables and Figure)

- **Supplementary Table 1: Results from liver function test (SuperChem).**
- **Supplementary Table 2: Results from complete blood count (CBC).**
- **Supplementary Table 3: Results from complete blood count (differential).**
- **Supplementary Figure S1. Histopathological organ analysis.**

Supplementary Table 1: Results from liver function test (SuperChem).

Shown are values from individual Balb/c mice (without xenotransplanted tumor cells). Three animals remained untreated; three animals received vehicle; three animals received 30 mg/kg NEO212; three animals received 60 mg/kg NEO212. Treatment was via subcutaneous injection once daily for 7 consecutive days. Eleven days after the end of the treatment cycle, animals were euthanized and blood was collected.

Blood samples were analyzed by SuperChem, a comprehensive blood chemistry panel that provides a good overview of many of the body's functions. Besides the liver function values summarized below, several additional physiological parameters were analyzed; however, no significant differences were found between the groups of NEO212-treated animals and those that did not receive the drug.

Component	Untreated	Vehicle	NEO212 30*	NEO212 60*
ALT (IU/L)	42<br>42<br>42	26<br>30<br>51	27<br>27<br>34	30<br>34<br>57
AST (IU/L)	98<br>118<br>162	68<br>120<br>369	84<br>84<br>180	76<br>138<br>144
ALP (IU/L)	86<br>104<br>126	69<br>75<br>90	84<br>84<br>90	72<br>74<br>108
Bilirubin (mg/dL)	0.2<br>0.2<br>0.3	0.1<br>0.2<br>0.3	0.2<br>0.3<br>0.3	0.2<br>0.2<br>0.3

* mg/kg

ALT: alanine transaminase; AST: aspartate transaminase; ALP: alkaline phosphatase.
All numbers are arranged by increasing values in each group.

Supplementary Table 2: Results from complete bloood count (CBC).

Shown are values from individual Balb/c mice (without xenotransplanted tumor cells). Three animals remained untreated; three animals received vehicle; three animals received 30 mg/kg NEO212; three animals received 60 mg/kg NEO212. Treatment was via subcutaneous injection once daily for 7 consecutive days. Eleven days after the end of the treatment cycle, animals were euthanized and blood was collected.

Component	Untreated	Vehicle	NEO212 30*	NEO212 60*
WBC (x10^3/uL)	1.5<br>1.9<br>2.1	1.5<br>1.5<br>2.5	3.2<br>4.0<br>5.6	1.6<br>2.0<br>2.9
RBC (x10^6/uL)	7.0<br>7.6<br>9.4	8.4<br>8.6<br>9.5	7.6<br>8.1<br>8.9	8.1<br>8.6<br>8.8
HGB (g/dL)	13.7<br>13.9<br>15.4	14.5<br>14.6<br>15.4	14.2<br>14.9<br>15.0	14.5<br>14.6<br>14.8
HCT (%)	43<br>46<br>55	48<br>51<br>55	46<br>48<br>52	49<br>51<br>51

* mg/kg

WBC: white blood cells; RBC: red blood cells; HGB: hemoglobin; HCT: hematocrit.
All numbers are arranged by increasing values in each group.

Supplementary Table 3: Results from complete blood count (CBC) with differential

Shown are values from individual Balb/c mice (without xenotransplanted tumor cells). Three animals remained untreated; three animals received vehicle; three animals received 30 mg/kg NEO212; three animals received 60 mg/kg NEO212. Treatment was via subcutaneous injection once daily for 7 consecutive days. Eleven days after the end of the treatment cycle, animals were euthanized and blood was collected.

Absolute numbers:

Component	Untreated	Vehicle	NEO212 30*	NEO212 60*
Neutrophils (x10^3/uL)	0.19<br>0.29<br>0.60	0.30<br>0.35<br>0.38	0.32<br>0.40<br>1.40	0.34<br>0.38<br>0.55
Lymphocytes (x10^3/uL)	0.75<br>1.63<br>1.76	1.05<br>1.10<br>1.95	2.53<br>3.36<br>3.42	1.17<br>1.56<br>2.12
Monocytes (per uL)	42<br>76<br>105	30<br>105<br>225	40<br>224<br>560	0<br>128<br>145
Eosinophiles (per uL)	0<br>0<br>45	0<br>0<br>25	96<br>200<br>224	0<br>60<br>87

Percent values:

Component	Untreated	Vehicle	NEO212 30*	NEO212 60*
Neutrophils (%)	10<br>14<br>40	12<br>23<br>25	10<br>10<br>25	19<br>19<br>19
Lymphocytes (%)	50<br>84<br>86	70<br>73<br>78	61<br>79<br>84	73<br>73<br>78
Monocytes (%)	2<br>4<br>7	2<br>7<br>9	1<br>7<br>10	0<br>5<br>8
Eosinophiles (%)	0<br>0<br>3	0<br>0<br>1	3<br>4<br>5	0<br>3<br>3

* mg/kg

All numbers are arranged by increasing values in each group.

Supplementary Figure S1. Histopathological organ analysis.

At the end of the in vivo experiment presented in Fig. 7, several animals from vehicle-treated and NEO212-treated groups were euthanized, and organs were collected for histopathological analysis. Organs were placed in 10% formalin for 24-48 hours. Thereafter, tissues were embedded in paraffin and cut into sections of 5 microns thickness. Slides were then stained using standard hematoxylin-eosin (H&E) stains, and evaluated under a light microscope. Representative photos are shown; no signs of organ damage could be detected in animals treated with NEO212.

Supplementary Figure S1

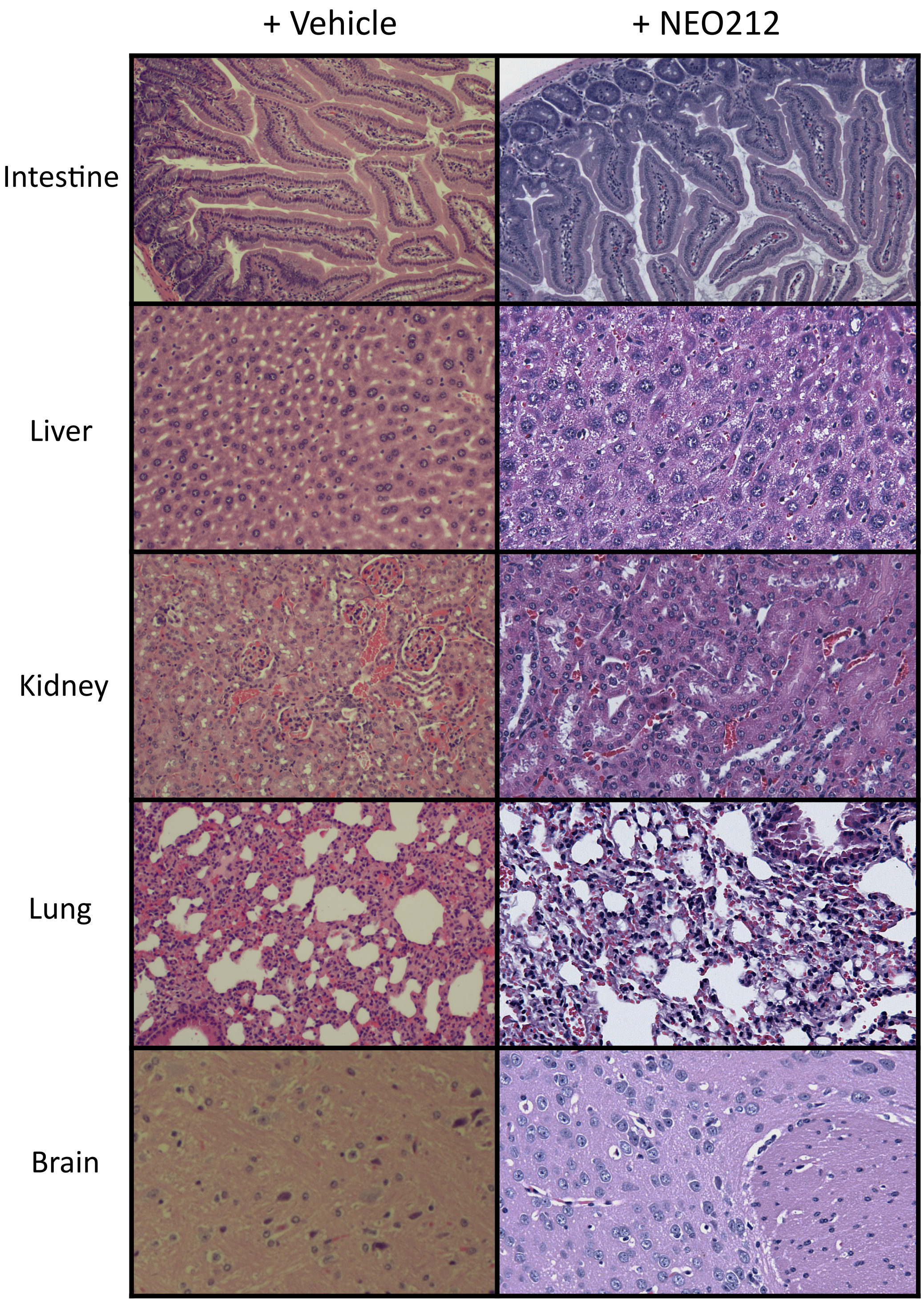

Supplement: Additional file 1: Table S1. — Results from liver function test (SuperChem). Table S2. Results from complete blood count (CBC). Table S3. Results from complete blood count (differential). Figure S1. Histopathological organ analysis. (PDF 6162 kb) [file 12929_2015_175_MOESM1_ESM.pdf]
